# Social learning in nest-building birds: a role for familiarity

**DOI:** 10.1098/rspb.2015.2685

**Published:** 2016-03-30

**Authors:** Lauren M. Guillette, Alice C. Y. Scott, Susan D. Healy

**Affiliations:** School of Biology, University of St Andrews, Harold Mitchell Building, St Andrews, Fife KY16 9TH, UK

**Keywords:** nest building, cognition, birds, social learning, physical cognition

## Abstract

It is becoming apparent that birds learn from their own experiences of nest building. What is not clear is whether birds can learn from watching conspecifics build. As social learning allows an animal to gain information without engaging in costly trial-and-error learning, first-time builders should exploit the successful habits of experienced builders. We presented first-time nest-building male zebra finches with either a familiar or an unfamiliar conspecific male building with material of a colour the observer did not like. When given the opportunity to build, males that had watched a familiar male build switched their material preference to that used by the familiar male. Males that observed unfamiliar birds did not. Thus, first-time nest builders use social information and copy the nest material choices when demonstrators are familiar but not when they are strangers. The relationships between individuals therefore influence how nest-building expertise is socially transmitted in zebra finches.

## 1. Introduction

Nest building is widespread across birds and other taxonomic groups. Wallace [1] notwithstanding, until recently nest building by birds was considered to be innate [2–5]. An accumulation of laboratory and field data, however, now point to limited repeatability in nest structure and to changes in material choice by nest builders that are dependent on the birds' own experience [6,7]. For example, zebra finches (*Taeniopygia guttata*) building in the laboratory learn to associate the nest material they use with the success of that material: they can reverse their nest material preferences if they successfully fledge young [8] and they learn the structural properties of nest material, modifying both handling techniques [9] and increasingly choosing more efficacious material [6].

Not only do birds learn about building materials by trial-and-error learning, but it also seems plausible that a builder would benefit from capitalizing on the success of others' nest-building experiences. Indeed, because some birds use social information to decide where to build their nest [10–13], they might also use social information to learn how to build a nest, including for example which material to use and how to handle that material.

Individuals should not copy indiscriminately, however, but should do so only in those situations where social learning is more beneficial than trial-and-error learning [14]. One situation in which it might pay to copy others is when individuals are uncertain [15], as is the case for birds that have no nest-building experience. To determine, then, whether first-time builders use socially provided information, we gave a first-time builder (the observer) and his mate the opportunity to watch an experienced nest-builder (the demonstrator) choosing to build a nest with material of one colour that the observers did not like, but not of another that was the observer's preferred colour. After this experience, we allowed the observers to build their first nest. In this way, we did not manipulate the functional outcome of the demonstrator's nest-material choice; we tested whether arbitrary social information (i.e. the colour of the material) would be transmitted from experienced to first-time nest builders. If inexperienced builders learn from conspecifics, then first-time builders should take advantage of the opportunity to learn which material to choose from an experienced builder.

In some species, however, directed social learning of physical skills has been reported: in brown capuchin monkeys (*Cebus apaella*), young, inexperienced individuals copied older individuals who are proficient at nut cracking [16,17], whereas in ravens (*Corvus corax*), transmission of social information (manipulating a target object) between individuals occurred more often in individuals with strong affiliative relationships (siblings and pair-bonded individuals) [18,19]. In zebra finches, which, like corvids, are both gregarious and form lifelong pair bonds, the identity of conspecifics may be relevant to social transmission of expertise. In the current experiment, we manipulated the provision of social information by presenting half the observers with a *familiar* builder, whereas the other half of the observers watched an *unfamiliar* individual build his nest. This allowed us to test two predictions. First, first-time builders use social information when learning what nest material to use. Second, first-timers are more likely to copy the choice of familiar nest builders than that of unfamiliar nest builders.

## 2. Methods

### (a) Subjects

The subjects in this experiment were 96 zebra finches bred at the University of St Andrews (23 female, 24 male), obtained from a local pet store (three female), obtained from Glasgow University (two female, four male) or obtained from breeders in Scotland (20 female, 20 male). All birds were housed in cages of same-sex individuals (8–30 individuals per cage, cage size 140 × 71 × 122 or 100 × 50 × 50 or 129 × 31 × 40 cm) and kept on a 14 : 10 light : dark cycle with temperature at approximately 20°C and humidity at approximately 50%. Birds were given free access to mixed seed, vitamin-supplemented water, cuttlefish bone, oyster shell and vitamin block. All birds were adults (at least 90 days post-hatch) at the time of testing.

All observers were naive with respect to building a nest, whereas the demonstrators had all previously built at least one nest. The female observers in the *unfamiliar* and all demonstrator birds in the *unfamiliar* group had been tested previously for preference of feeder colour [20], but none of the male observers had taken part in any experiments prior to this one.

### (b) Treatments

There were two treatments, *unfamiliar* and *familiar.* In the *unfamiliar* treatment, the demonstrators and the observers were not familiar (had no previous contact) with one another. Prior to and following testing, these observers and demonstrators were housed in separate colony rooms. In the *familiar* treatment, the demonstrators and the observers were familiar (had contact) with one another: for nine months prior to testing, all the males in this treatment were housed together in a single cage, whereas all of the females in the familiar group were housed together in a separate cage, in the same colony room.

### (c) Procedure

**Figure 1.**
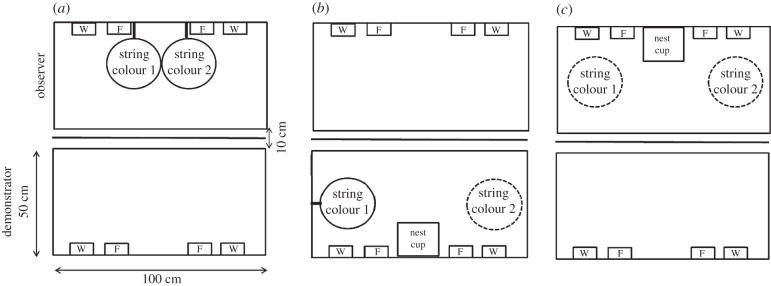
Schematic top-down view of experimental set-up during the different experimental phases. The observer cage is pictured at the top and the demonstrator cage is pictured at the bottom. W, water dish; F, food dish, provided ad libitum. (*a*) In the observer *initial colour preference*, the observer was given 25 pieces of pink and 25 pieces orange string that were attached to the front wall of the cage. (*b*) In the pre-observation phase, the demonstrator pair was given a nest cup and 50 pieces of the observer males preferred colour attached to the side wall of the cage (string colour 1) and 100 pieces of the observer males' least-preferred colour (string colour 2) to build with while the opaque barrier between the cages remained in place. In the observation phase, the demonstrator pair was given an addition 50 pieces of the observer males' least-preferred colour (string colour 2) to build with and the opaque barrier was removed. (*c*) In the test phase, the opaque barrier was returned and the observer pair was given 25 pieces of each coloured string and a nest cup.

We selected non-related individuals and paired birds by housing them in wooden cages with wire fronts (45 × 31 × 39 cm). Birds were paired for a minimum of 6 days before the experiment began, which allowed for adequate time to form a pair bond [6,21]. Between 14.30 and 15.30 on the day before testing commenced, one observer pair and one demonstrator pair were moved to the experimental room. The experimental room contained two cages (100 × 50 × 50 cm) that faced each other along the 100 cm side of the cages, at a distance of 10 cm (figure 1). A white opaque barrier between the cages prevented visual, but not vocal, interaction between the observer and demonstrator pairs. Each cage contained two water bowls, two food bowls, a cuttlefish bone, oyster shells, a vitamin block (to which birds had ad libitum access throughout the experiment) and six perches. Each cage also had three bird-box cameras suspended from the ceiling of the cage so that all behaviour was recorded (SpyCameraCCTV, Bristol, UK) on a laptop computer. Two different colours of string (orange and pink; jute craft twine, James Lever Co., London, UK) were used in testing.

Each observer male was run in one trial, and each trial consisted of four phases: (i) observer initial colour preference, (ii) demonstrator pre-observation building, (iii) observation and (iv) observer test phase.

#### (i) Observer initial colour preference

The male in each observer pair was tested for his preference of the two different colours of string (pink and orange) to be used in the subsequent experimental phases (figure 1*a*). At 09.00 on day 1 of the experiment, we placed 25 pieces of pink string and 25 pieces of orange string (each 15 cm long) into the observers' cage. Each piece of coloured string was tied in a bundle to the front of the cage. This allowed the observers to manipulate the coloured string, but not to carry it away or use it to build a nest.

We then recorded the observers' behaviour for 2 h. After 2 h, we removed all string from the observers' cage and scored the video immediately to measure the amount of time the male had interacted with each colour of string. If the male had spent less than 60 s interacting with the string, then we returned string of both colours to the observers' cage and recorded their behaviour for a further 2 h of recording. After these 2 h, we again removed all the string from the cage and scored the video. If after this second, then 2 h session the male had still spent less than 60 s in total interacting with the string, we returned string of both colours to the observers' cage for a third and final 2 h recording. If, after any of the 2 h sessions, the male had spent at least 60 s interacting with the string, then we calculated his preference for string colour by dividing the amount of time he had spent interacting with the colour of string with which he had spent the most time interacting by the total amount of time he had spent interacting with both colours of string. Therefore, the maximum time allowed for the *observer initial colour preference* phase was 6 h (i.e. up to three session that were 2 h each). The minimum time allowed was 2 h. We excluded those males that, after the full 6 h, had spent less than 30 s interacting with the coloured string from the experiment (*n* = 3 pairs, 2 in the *unfamiliar* group, 1 in the *familiar* group). As soon as we had assessed the male's preference for string colour, the second phase of the experiment started.

#### (ii) Demonstrator pre-observation building

This phase commenced immediately after we had determined the observer males' string colour preference. We gave the demonstrator pair 100 pieces (15 cm each) of the observer male's least-preferred colour string. These 100 pieces were placed against one of the side walls of the demonstrators' cage and were sham tied to the cage. On the opposite side of the demonstrators' cage, we then placed 50 pieces of the observer's preferred colour string against the cage wall. These pieces were, however, actually tied to the cage wall. This set-up allowed the demonstrators to interact with string of both colours, but the male could carry away and build a nest only with the observer male's least-preferred colour. By having both colours of string (the observers preferred and non-preferred) present during the *observation phase*, we can attribute a preference change to the demonstrated colour during the *test phase* as a result of social demonstration and not exposure to the demonstrated string. We hung a wooden nest cup (11 × 12 × 4.5 cm) on the wall of the long side of the demonstrator cage that was opposite the white opaque barrier separating the demonstrator and observer cages. The white opaque barrier was in place for the duration of this phase, so the observer and demonstrator pair had no visual contact with one another. This phase lasted until the demonstrator male had taken all 100 pieces of string to the nest cup (figure 1*b*). The purpose of this phase was to ensure that the demonstrator was engaged in nest building before the observation phase began.

#### (iii) Observation

**Figure 2.**
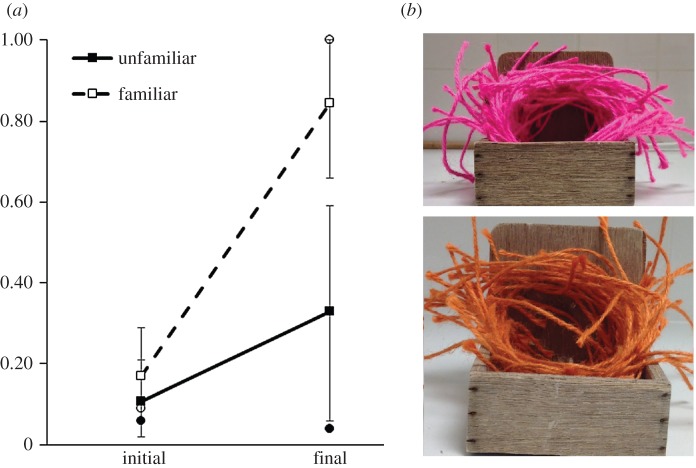
(*a*) The initial and final preferences tests and the proportion of demonstrated colour for birds in the *unfamiliar* (filled markers) and the *familiar* group (open marker). The squares represent the group mean and the 95% CI, and the circles represent the median score. The dependent measure in the first preference test is the time observers spent interacting with tied-down string of two colours (pink and orange). Males then watched demonstrators build a nest with the observer's unpreferred colour. The dependent measure in the final preference test was the proportion of demonstrated colour brought to the nest cup by the observer male. (*b*) Photographs of demonstrators' nests after the demonstration phase. Each of these nests was built with 150 pieces of 15 cm string. (Online version in colour.)

In this phase, the demonstrator pair received an additional 50 pieces of the same colour string with which they had built in the previous phase to add to the partially constructed nest (100 pieces). The 50 pieces of the observers initially preferred colour from the *demonstrator pre-observation building* remained in the demonstrator cage, secured to the wall. We removed the white opaque barrier between the demonstrator and observer cage, so that the pairs now were in visual contact with one another. The observation phase lasted until the demonstrator male added the additional 50 pieces of string to the nest. The cut-off for this phase of the experiment was 3 days. If the demonstrators had not added the additional 50 pieces of string to the nest by this time, the birds were removed from the experiment (*n* = 2 pairs in the *unfamiliar* group). See figure 2 for a picture of nests constructed with 150 pieces of material.

#### (iv) Observer test

The observer test began once the *observation* phase was complete. We returned the opaque barrier, so that the observer and demonstrator pairs were no longer in visual contact. We then placed 25 pieces of pink and 25 pieces of orange string (15 cm each) into the observers' cage, in the same position as the string in the *observation* phase (figure 1*c*). The placement of each colour mirrored that of the demonstrator cage. We hung a wooden nest cup (11 × 12 × 4.5 cm) on the wall of the long side of the observer cage that was opposite the white opaque barrier that separated the demonstrator and observer cages. The test phase ended once the entirety of one pile of string had been added to the nest cup.

### (d) Scoring

We assessed the *final colour preference* of the observers as the proportion of string of the first 25 pieces the male observer had deposited in the nest cup that were of the demonstrated colour. This score was therefore 0 if the first 25 pieces the observer took to build his nest were of his initially preferred colour, the score was near 0.5 if the male had incorporated an equal number of pieces of his initially preferred colour as of the demonstrated colour, whereas it was near to 1 if the male did not use the material that he had initially preferred but rather built with nest material of the same colour as that with which the demonstrator had built.

### (e) Statistical analyses

We conducted one-sample Wilcoxon signed-rank tests with the chance level of 0.5 (using both colours equally) on the *final colour preference* to test for systematic copying in the two treatment groups. We used a Mann–Whitney *U*-test to test for differences between the two treatment groups (one test for the *initial*, and a second test for the *final colour preference*). We report the median proportion and upper and lower level of the 95% CI that were bootstrapped and bias corrected (1000 samples). We also used a related-sample Wilcoxon test to test for a change in colour preference between the *initial* and *final colour preference* within each treatment group*.* Three pairs (two in the *unfamiliar* group, one in the *familiar* group) were removed from the experiment after the initial colour preference phase, because the males did not spend at least 30 s interacting with the coloured string. Two pairs of demonstrators (both in the *unfamiliar* group) stopped building in the observation phase. One pair of observers in the *familiar* group did not build a nest. The final sample sizes were *n* = 10 in the *unfamiliar* and *n* = 8 in the *familiar* group.

## 3. Results

### (a) Observer choice after social demonstration

**Figure 3.**
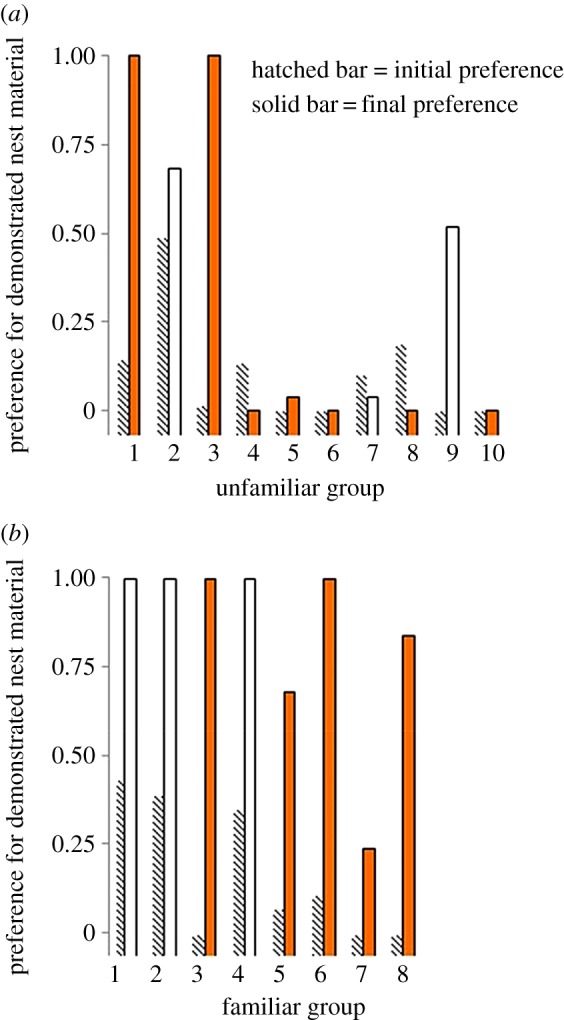
The preference for the colour of nest material demonstrated to males in the (*a*) *unfamiliar* and (*b*) *familiar* treatment groups. Each pair of bars represents one male. The colour of the bar represents the demonstrated colour (open bar, pink; filled bar, orange—which was the initially unpreferred colour for each male). The hatched bar is the initial preference for the demonstrated colour and the solid bar is the final preference for the demonstrated colour. (Online version in colour.)

Prior to observing the demonstrators build a nest, the first-time builders' preference for the demonstrated colour of nest material did not differ between the birds in the *familiar* (*n* = 8, median = 0.09, lower = 0.06, upper = 0.29 95% CI) and those in the *unfamiliar* treatments (*n* = 10, median = 0.06, lower = 0.02, upper = 0.21; independent-samples Mann–Whitney, *U*_18_ = 0.366 *p* = 0.76). After the building demonstration, however, the birds in the *familiar* treatment (median = 1.0, lower = 0.66, upper = 1.0 95% CI) had a stronger preference for the demonstrated colour material than birds in the *unfamiliar* treatment (median = 0.04, lower = 0.08, upper = 0.59; independent-samples Mann–Whitney, *U*_18_ = 2.44, *p* = 0.02; figure 2). All birds (*n* = 8) in the *familiar* group increased their preference for the demonstrated colour material in the final colour preference test (related-samples Wilcoxon test: *W* = 2.524, *n* = 8, *p* = 0.01), but the birds in the *unfamiliar* group did not (related-samples Wilcoxon test: *W* = 1.260, *n* = 10, *p* = 0.21; figure 3). Furthermore, after the demonstration, the material colour preference of the birds in the *familiar* group was significantly greater than chance (Wilcoxon signed-rank test: *W* = 2.3, *n* = 8, *p* = 0.02), whereas that of the birds in the *unfamiliar* group was not (Wilcoxon signed-rank test: *W* = −0.99, *n* = 10, *p* = 0.32).

We also quantified which colour of nest material was first touched, picked up and deposited into the nest cup by the observer males when tested after the building demonstration. In the *unfamiliar* group of observers, seven of 10 birds first touched the demonstrated colour, six of them picked it up first, but only three of them deposited this material into their nest cup before they deposited any of the non-demonstrated colour material. These data show that birds that were not familiar with the demonstrators did indeed pay attention to the demonstrators' behaviour, but that they disregarded this information when they came to build a nest of their own. In the *familiar* group, five of eight birds touched the demonstrated colour, seven picked up the demonstrated colour and five deposited this material into their nest cup before they deposited any of the non-demonstrated colour material (see electronic supplementary material).

## 4. Discussion

Male zebra finches building their first nest used social information to guide their choice of nest material: first-time builders copied the material choice of familiar individuals. For our first-time builders, the identity of the watched bird was therefore important. This selective transfer of knowledge between some individuals, but not others, is a feature that promotes the formation and maintenance of cultural traditions [22–25]. For example, just as our birds learned which material to use based on its colour, so vervet monkeys (*Chlorocebus aethiops*) copy the colour choice of others feeding on pink or blue maize corn [23], and great tits (*Parus major*) copy the choice of door based on its colour (blue or red [25]). In both the vervets and great tits, the colour preference of the initial demonstrators became the colour preferred by the majority even when individuals experienced success with the alternative colour [25] or moved to a different group of animals eating food of the other colour [23]. In this study, we provide evidence that birds copy colour preferences for nest material expressed by familiar, but not unfamiliar, individuals even when this preference confers no mechanical advantage in terms of the final structure of that nest. While the studies with the vervets and tits show that potential emergence of cultural though conforming to the majority, our results show that conformity may begin among familiar individuals. These examples from both the wild and the laboratory show copying among individuals for arbitrary features, which may be analogous to human trends and fashion.

These data show that first-time nest-building birds learn to select nest material based on one physical property (colour) from watching a familiar male build a nest. Attending to, and selecting, material based on its physical properties is crucial to both the building of a nest and to the making and using of tools. Some animals, including humans [26], can learn how to use tools from watching others do so (e.g. chimps, *Pan troglodytes* [27,28]; capuchin monkeys [17]; bottlenose dolphins, *Tursiops* sp. [29]). This does not seem to be the case, however, for birds that make and use tools in the wild. Tool-using woodpecker finches do not learn how to modify or to use twigs or cactus spines to forage for insects in tree holes from watching others [30]. Similarly, New Caledonian crows (*Corvus moneduloides*) manufacture and use tools in the absence of social demonstration [31] and although observations from the field show there are ample opportunities for juvenile crows to learn how to use tools from watching conspecifics [32,33], there is no evidence to suggest that they do. The Goffin cockatoo (*Cacatua goffini*), on the other hand, can learn how to make tools in a captive aviary setting from watching a conspecific [34], although they do not make or use tools in the wild. The suggested mechanism was *result emulation* (*sensu* [30]), where the observer reproduces the result of the demonstrator's actions without attributing a goal to the demonstrator's behaviour. One way to tease apart and understand the mechanism(s) underlying social acquisition of tool use and other physical skills, including nest building, would be to provide observers with several demonstrators, each using a different technique that leads to a different outcome.

Nest building, then, is a system that is amenable to experimental manipulation to examine such mechanisms underlying the transmission of information about physical skills. Together with data that show that zebra finch males learn about the structural properties of material [6] and how to modify their material handling techniques [9], our current data suggest that nest building may be one aspect of physical cognition that birds can learn socially in the wild. Future work on nest building can now focus on whether observers might learn how to manipulate nest material, to choose more or less appropriate materials and so on from watching experienced builders. In contrast with the paucity of species that make and use tools, nest building is both ubiquitous and variable among birds. This variability comes in several ways; for example, in terms of the effort expended on material selection, material handling/manipulation/removal, or the final structure of and who builds the nest (e.g. male only, female only, both, communal; see [35] for a review). Nest-building behaviour in birds appears, then, to be a useful system for examining not only the evolutionary and ecological roots of physical cognition, but also cultural transmission and conformity.

## Supplementary Material

Supplementary Material

## Data accessibility

All data are archived in the electronic supplementary material for this article.
