# Supplementary material for: Social learning in nest-building birds: a role for familiarity
Source: Proc Biol Sci. 2016 Mar 30;283(1827):20152685. doi: 10.1098/rspb.2015.2685 (PMC4822453; doi:10.1098/rspb.2015.2685)
Supplement: Supplementary Material [file rspb20152685supp1.pdf]

Supplementary Material Guillette et al.

Supplementary Figure 1. The proportion of males in the *unfamiliar* group (black bars n=10) and the *familiar* group (grey bars n=8) that interacted with the demonstrated colour nest material before they interacted with nest material of their initially preferred colour. Touch refers to the observer male touching the nest material with his beak or foot. Pick up refers to the holding by a male of the nest material in his beak and removing it from the pile of nest material. Deposit refers to the first string that a male put into the nest cup. Copy refers to those males with a final colour preference of >0.5 for the demonstrated colour nest material.

Supplementary Figure 2. The preference for the colour of nest material of inexperienced males before and after these males watched an experienced bird building. Observers that watched *unfamiliar* builders are shown in Panel a (squares) while the observers that watched *familiar* builders are shown in Panel b (triangles). The lines denote the change in preference for a single male.

Supplementary Table 1. The raw data for colour preferences for all pairs of observers. The *initial colour preference* was the number of seconds during which the male interacted with the different coloured string. The *final colour preference* was the number of pieces of string of each colour in the first 25 nest deposits, u = unfamiliar and f = familiar treatment groups.

Supplementary Figure 1.

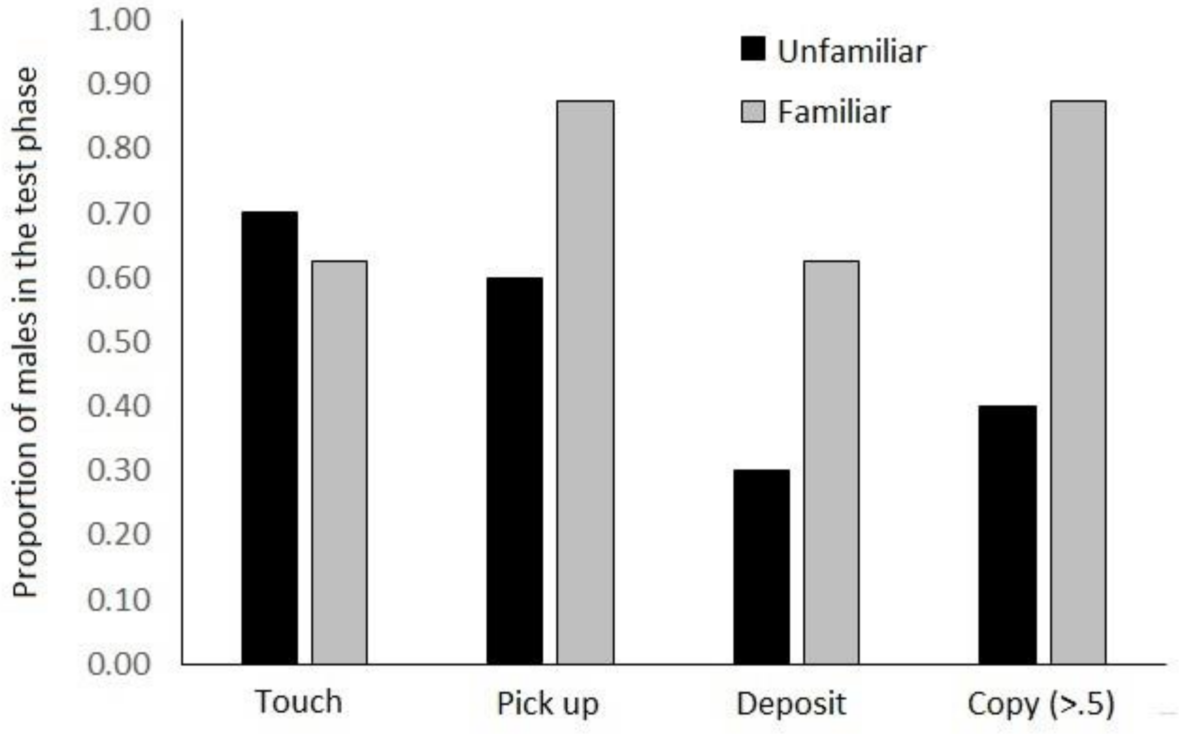

Supplementary Figure 2.

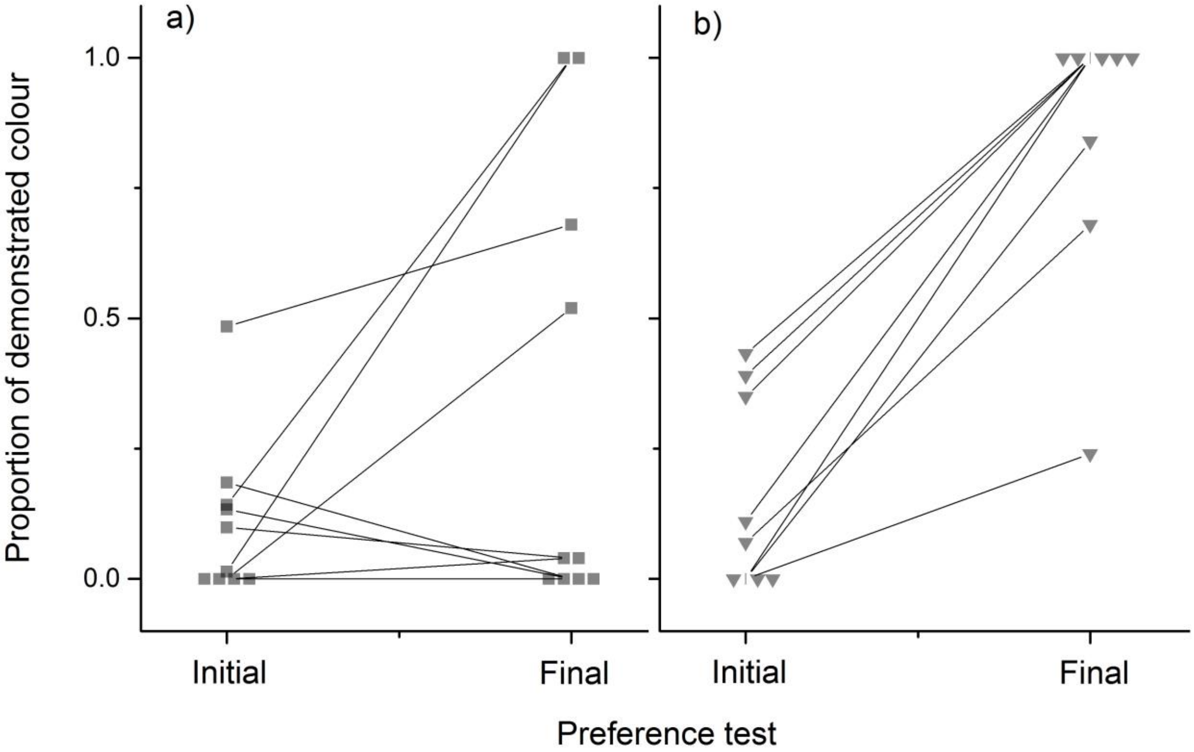

Supplementary Table 1.

		Initial colour preference: time (s)		Final colour preference: number of pieces deposited		
Pair	Group	Non-demonstrated colour	Demonstrated colour	Non-demonstrated colour	Demonstrated colour	Demonstrated colour
2	u	96.02	15.96	0	25	orange
3	u	804.28	755.25	8	17	pink
5	u	458.38	6.68	0	25	orange
6	u	63.44	9.78	25	0	orange
7	u	52.108	0.00	24	1	orange
9	u	132.50	0.00	25	0	orange
10	u	1250.00	137	24	1	pink
11	u	25.14	5.71	25	0	orange
13	u	69.29	0.00	12	13	pink
14	u	66.408	0.00	25	0	orange
15	f	44.21	33.62	0	25	pink
16	f	57.11	36.93	0	25	pink
17	f	1291.00	0.00	0	25	orange
18	f	281.00	149.00	0	25	pink
19	f	292.00	22.00	8	17	orange
20	f	129.00	16.00	0	25	orange
21	f	82.00	0.00	19	6	orange
24	f	2113.00	0.00	4	21	orange
